# The Effects of Multiple Global Change Factors on Soil Nutrients across China: A Meta-Analysis

**DOI:** 10.3390/ijerph192215230

**Published:** 2022-11-18

**Authors:** Xinyi Shen, Junwei Ma, Yuqian Li, Yijia Li, Xinghui Xia

**Affiliations:** 1 State Key Laboratory of Water Environment Simulation, School of Environment, Beijing Normal University, No. 19 Xinjiekouwai Street, Haidian District, Beijing 100875, China; 2 State Key Laboratory of Environmental Criteria and Risk Assessment, Chinese Research Academy of Environmental Sciences, Beijing 100012, China

**Keywords:** global change factor, individual and combined effect, interactive effect, additive interaction, soil nutrient

## Abstract

The quantification of the effects of global changes on soil nutrients is crucial for the prediction of future terrestrial ecosystem changes. Combined with 100 articles and 1129 observations from all over China, the meta-analysis method was applied to explore the effects of various global change factors on soil nutrients, including precipitation change, nitrogen addition, warming, and carbon dioxide (CO_2_) concentration rise. Results indicated that among all the individual drivers, soil nutrients are most sensitive to N addition. Significant positive effects of N addition on carbon concentration (+4.6%), nitrogen concentration (+6.1%), organic carbon (+5.0%), and available nitrogen (+74.6%) were observed considering all the land-use types. The results highlighted that the combined and interactive effects of multiple global change factors on soil nutrients were of great significance. The interaction of the two drivers is usually additive, followed by antagonism and synergy. Our findings contribute to better understanding of how soil nutrients will change under future global change.

## 1. Introduction

The quality and supply of soil nutrients have important implications for global biochemical cycles [1,2]. Carbon (C), nitrogen (N), phosphorus (P), and potassium (K) are critical elements in ecosystems and might be limitation nutrient resources restricting plant growth and production [3]. Their concentrations and availability in soils are identified as important indicators of soil nutrients [4], and also play a crucial role in ecosystem structure and functioning by affecting microbial community structure and biodiversity and by altering plant species and diversity [5,6,7,8]. Soil nutrient concentrations and availability are controlled by parent materials [9], microbial activity [10], anthropogenic factors [11], and global changes [12].

Apart from the global atmospheric CO_2_ rise, China has been intensely affected by warming [13,14], altered precipitation [15,16], and elevated N deposition [14] in the last decades. Over the past 50 years, the country-averaged annual surface mean temperature has increased by 1.1 °C and is anticipated to warm at similar rates throughout the remainder of this century [17]. The average precipitation changes showed geographical variations, such as increased precipitation observed during the last 30 years in arid and semiarid regions, but showed no uniform pattern [18]. Without explicit additional efforts to constrain emissions, the CO_2_ concentration will exceed 450 ppm by 2030 and 750–1300 ppm by 2100 [19]. The average N deposition has increased rapidly in China from 1.32 kg N ha^−1^ (1980s) to 21.1 kg N ha^−1^ (2000s), and the growth is expected to continue [20].

Global change drivers may profoundly affect biogeochemical cycling in terrestrial ecosystems, which has been studied extensively, mainly involving the effect of an individual driver or a pair of two drivers. Temperature or precipitation changes motivate the decoupling and imbalance of nutrients by changing the soil properties, moisture levels, respiration levels, plant biomass and productivity, and microbial activity [21,22,23,24,25]. Elevated CO_2_ concentration could increase the net primary productivity and C storage [26,27,28]. It is important to individually assess the responses of soil nutrients to global change drivers. However, the effects of a single driver might be meditated by other factors. Elevated atmospheric CO_2_ concentration and N deposition may stimulate the plant to absorb nutrients by promoting plant growth [29,30], while other research found that N deposition might increase the soil acidity, inhibit soil respiration, and lead to the accumulation of some soil nutrients [31]. The multiple global change drivers often act simultaneously at large spatial and time scales. This indicated that the combined effects of multiple global drivers may be of greater importance than individual effects [32]. Moreover, the interaction of global change factors pairs on soil nutrients are still unknown. Is the interaction additive, antagonistic, or synergistic?

Meta-analyses have been performed on the effects and interactions of global change factors on soil properties, involving soil respiration [33], P pools [34], and C pools [25]. There were few syntheses so far for the individual effects and the interaction of the global change factors on soil nutrients. The evaluation of soil nutrients is relatively lacking, so it is difficult to accurately predict whether the interaction of multiple global change factors on soil nutrients is additive or not. Previous studies suggest that the magnitude of ecosystem response to global change drivers may decline with higher-order interactions (i.e., the combined effects of two or more global change drivers) and at larger spatial scales [35]. This would mean that either positive or negative effects of individual factors on ecological stoichiometry and ecosystem functioning will be more significant than combined effects. Including logistical limitations, we only consider the effects of single factors and two-driver pairs here. The results will help predict future changes and functions and can be incorporated into ecosystem models and biogeochemical cycles under future global change scenarios.

Therefore, we conducted a meta-analysis with the results from 100 papers and 1129 observations to explore the effects of multiple global change factors, including warming, altered precipitation, elevated CO_2_ concentration, and elevated nitrogen addition on soil nutrients across different ecosystems in China. We hypothesize that (i) the individual effect of a global change driver on soil nutrients is stronger than when combined with another driver, (ii) the interactive effects of two global change drivers will be additive rather than synergistic or antagonistic, (iii) the response of soil nutrients to global change drivers significantly vary among ecosystems, and (iv) soil nutrients’ responses to global change can be significantly affected by environmental or experimental conditions including climate, geographical locations, and experimental durations.

## 2. Methods and Materials

### 2.1. Data Collection

We used China National Knowledge Infrastructure (CNKI) and Web of Science to search the primary published studies (before July 2022) in Chinese and in English that evaluated the individual and combined effects of either elevated CO_2_ concentration (C), warming (W), reduced precipitation (RP), elevated precipitation (EP), or N deposition (N) and their interaction on soil nutrients, including the total carbon (TC), soil organic carbon (SOC), total nitrogen (TN), available nitrogen (AN), total phosphorus (TP), available phosphorus (AP), total potassium (TK), and available potassium (AK) of terrestrial ecosystems in China.

To establish a comprehensive experimental database on the effects of global change factors on soil nutrients in terrestrial ecosystems in China, experiments must meet our following criteria to be entered in our database: (1) the experiments contained at least one of the target soil nutrient responses to at least one of the mentioned global change factors; (2) at least 2 × 2 full factor field experiments were conducted, which simultaneously considered controls in the same ecosystem and under the same environmental conditions; (3) the experimental conditions and treatments were clearly recorded; the sample sizes, mean values, and standard deviations (or standard errors) of both the experimental and control groups could be extracted from the tables, figures, or context; and (4) experiments data from only the topsoil layer (<20 cm) in terrestrial ecosystems.

**Figure 1:**
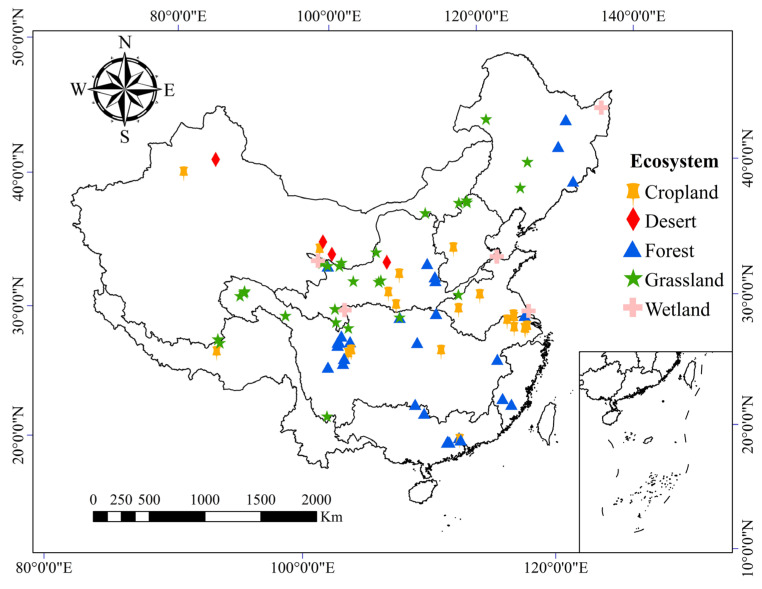
Locations of the experiment sites among different ecosystems included in this meta-analysis.

In addition, to explore the influence of the environmental conditions and experimental factors, we also recorded the site characteristics. Site characteristics included site location (latitude and longitude), climate variables (MAT and MAP), ecosystem type, and experimental duration for each publication. In total, 1129 observations were collected, including 933 individual observations and 196 combined observations from 88 sites and from different ecosystems (Figure 1, Supplementary Materials Dataset).

### 2.2. Data Analysis

#### 2.2.1. Individual and Combined Effects

We quantified the effects of global change factors (both the individual effects of one factor and the combined effects of factor pairs) on soil nutrients by weighting the natural log-transformed effect size (*lnRR*) with the inverse variance and a random-effect model [36]. The response ratio (*RR*) is defined by the ratio of the mean value of soil nutrients of the treatment with climate factors changed ($\bar{X_{e}}$) to that of the control treatment ($\bar{X_{c}}$) (Equation (1)). The *lnRR* was used to reduce bias and to ensure a normal sampling distribution, as described by [37].

(1) $$lnRR=ln\left(\bar{X_{e}}/\bar{X_{c}}\right)=ln\bar{X_{e}}-ln\bar{X_{c}}$$

With a variance of:

(2) $$V_{lnRR}=V_{i}+T^{2}$$

$V_{i}$ is the within-study variance of study. The $V_{i}$ was calculated as follows:

(3) $$V_{i}=S_{e}^{2}/\left(n_{e}\bar{X_{e}^{2}}\right)+S_{c}^{2}/\left(n_{c}\bar{X_{c}^{2}}\right)$$

*T*^2^ is the between-studies variance. The calculation of *T*^2^ can be seen in Borenstein et al. [38].

In the equation above, $\bar{X_{e}}$,$\;n_{e}$, and $S_{e}$ refer to the mean values, the sample sizes, and the standard deviations (SD) of the concerned variable in the treatment groups, respectively; $\bar{X_{c}}$,$\;n_{c}$, and $S_{c}$ are the mean values, the sample sizes, and the standard deviations (SD) of the control groups, respectively. If standard error (SE) rather than SD was reported, then SE was first converted into SD.

The random-effects model was selected because the main assumptions are more likely to be satisfied when dealing with ecological data synthesis and they count more heavily those studies with more replication and less variance [39]. The weighted response ratio ($lnRR_{++}$) was calculated using Equation (4):

(4) $$lnRR_{++}=\left(\sum_{i=1}^{m}\sum_{j=1}^{k}w_{ij}lnRR_{ij}\right)/\left(\sum_{i=1}^{m}\sum_{j=1}^{k}w_{ij}\right)$$

where *m* is the number of groups, *k* is the number of experiments in the group (e.g., an ecosystem), and *w* is the weighting factor of the *j*th experiment in the group. The weighting factor could be expressed as the reciprocal of their variance (1/V*_lnRR_*) to account for differences in precision among experiments. The 95% confidence interval (CI) for each effect was generated by bootstrapping tests with 999 iterations, and effects were significant (*p* < 0.05) if the 95% CI did not overlap with 0 [40]. The percentage change in soil nutrients induced by the considered global change factor was calculated as follows:

(5) $$Mean\;percentage\;of\;the\;change=\left(e^{lnRR_{++}}-1\right)\times 100\%$$

#### 2.2.2. Interactive Effects

The interactive effect of the global change factors on soil nutrients were evaluated using Hedges’ *d*. The individual Hedge’s *d* of a factor was calculated using Equation (6):

(6) $$d=[\left(\bar{X_{e}}-\bar{X_{c}})/s\right]\cdot J\left(m\right)$$

where *s* and *J*(*m*) are the pooled standard deviation and correction term for small sample bias, respectively, which were estimated by Equations (7) and (8), respectively:

(7) $$s=\sqrt{\left[\left(n_{e}-1\right)S_{e}^{2}+\left(n_{c}-1\right)S_{c}^{2}\right]/\left(n_{e}+n_{c}-2\right)}$$

(8) J(m)=1−3/(4m−1)

*m* is the degree of freedom ($m=n_{e}+n_{c}-2$).

The variance ($V_{I}$) of the individual *d* was estimated by Equation (9):

(9) $$V_{I}=\left(n_{e}+n_{c}\right)/n_{e}n_{c}+d^{2}/\left[2\left(n_{e}+n_{c}\right)\right]$$

The weight ($W_{I}$) was the reciprocal of the variance (i.e., $1/V_{I}$), which was used to calculate the weighted *d* ($d_{++}$ in Equation (10) and standard error ($s\left(d_{++}\right)$ in Equation (11):

(10) $$d_{++}=\left(\sum_{i=1}^{l}\sum_{j=1}^{k}w_{ij}d_{ij}\right)/\left(\sum_{i=1}^{l}\sum_{j=1}^{k}w_{ij}\right)$$

(11) $$s\left(d_{++}\right)=\sqrt{1/\left(\sum_{i=1}^{l}\sum_{j=1}^{k}w_{ij}\right)}$$

where l is the number of the groups and k is the number of the comparisons in the *i*th group.

The main effects pf global change factors A and B ($d_{A}$ and $d_{B}$) and their interaction ($d_{AB}$) are shown in Equations (12)–(14):

(12) $$d_{A}=\left\{\left[(X_{A}+X_{AB}\right)-\left(X_{B}+X_{C}\right)]/2s\right\}\cdot J\left(m\right)$$

(13) $$d_{B}=\left\{\left[(X_{B}+X_{AB}\right)-\left(X_{A}+X_{C}\right)]/2s\right\}\cdot J\left(m\right)$$

(14) $$d_{AB}=\left\{\left[(X_{AB}+X_{A}\right)-\left(X_{B}+X_{C}\right)]/2s\right\}\cdot J\left(m\right)$$

where s and J(m) are the pooled SD and correction term for small sample bias, respectively, which were estimated by Equations (15) and (8), respectively:

(15) $$s=\sqrt{\left[\left(n_{C}-1\right)S_{C}^{2}+\left(n_{A}-1\right)S_{A}^{2}+\left(n_{B}-1\right)S_{B}^{2}+\left(n_{AB}-1\right)S_{AB}^{2}\right]/\left(n_{c}+n_{A}+n_{B}+n_{AB}-4\right)}$$

The degree of freedom (m) here used in Equation (8) is $m=n_{c}+n_{A}+n_{B}+n_{AB}-4$. The variance of Hedges’ d of the main or interactive effects was estimated by Equation (16):

(16) $$V_{d_{t}}=\left\{1/n_{C}+1/n_{A}+1/n_{B}+1/n_{AB}+d_{t}^{2}/\left[2\left(n_{c}+n_{A}+n_{B}+n_{AB}\right)\right]\right\}/4$$

where t is the treatment of A, B, or A + B. The weight ($W_{dt}$) was also reciprocal of variance as before; the weighted mean $d_{t}$ ($d_{t++}$) and standard error were calculated according to Equations (10) and (11).

In the equation above, $X_{C}$, $n_{C},$ and $S_{C}$; $X_{A}$, $n_{A},$ and $S_{A}$; $X_{B}$, $n_{B}$, and $S_{B}$; and $X_{AB}$, $n_{AB}$, and $S_{AB}$ are the mean value, sample size, and SD in the control group, treatment *A*, treatment *B*, and their combination (i.e., *A* + *B*), respectively.

Accordingly, the type of interaction between the two factors was classified into additive, synergistic, and antagonistic [24]. If the 95% confidence interval overlaps with zero, the interaction effect is considered as an additive effect. Otherwise, if the individual effect is negative or the opposite direction of the two-driver pairs, the positive interaction effect represents antagonism, and the negative interaction effect represents synergy. For two-driver pairs where both interaction effects are positive, positive interaction effects represent synergy, while negative interactions represent antagonism.

### 2.3. Statistical Analysis

The original data were obtained by means of charts and supplementary materials in addition to the texts of the selected studies. If only figures were given, we used version 2.26 of the GetData Graph Digitizer (http://getdata-graph-digitizer.com/, accessed on 31 July 2022.) to extract the data. The weighted response ratio ($lnRR_{++}$) and 95% confidence intervals (CI) were calculated using the random-effects model in MetaWin 2.1 (Sinauer Associates Inc., Sunderland, MA, USA). The relationships between the *lnRR* of the soil nutrient and moderator variables (i.e., the longitude, latitude, experimental duration, mean annual precipitation (MAP), and mean annual temperature (MAT)) under the individual and combined effects of the global change factors were assessed by conducting linear regression analyses with SPSS 25.0 (SPSS Inc., Chicago, IL, USA).

## 3. Results and Discussion

### 3.1. Individual Effects of Single Global-Change Factor on Soil Nutrients

#### 3.1.1. Effects of Elevated CO_2_ Concentrations

**Figure 2:**
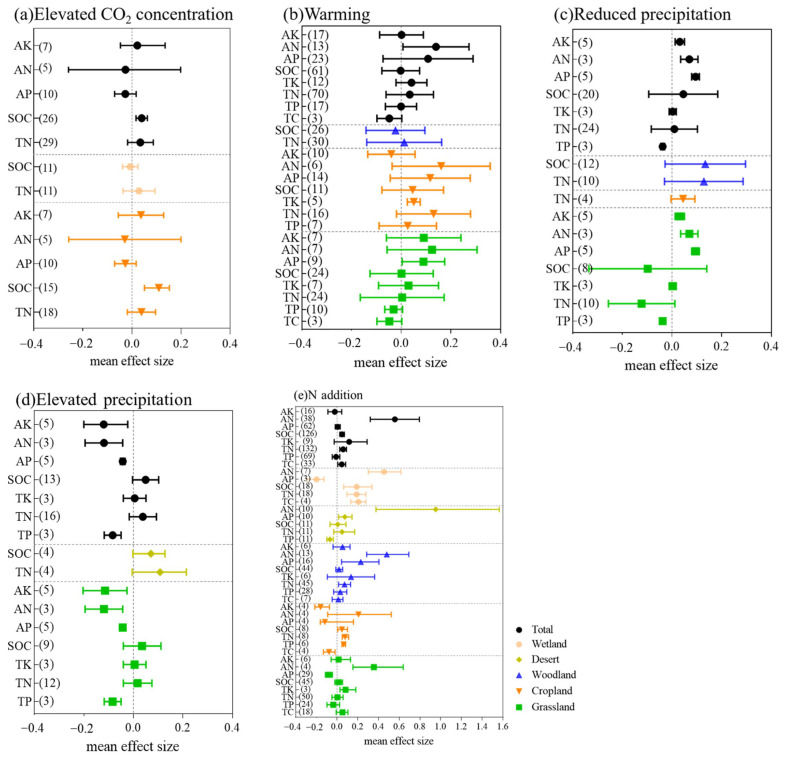
Individual effects of climate change factors on soil nutrients. (**a**) Elevated CO_2_ concentration, (**b**) Warming, (**c**) Reduced precipitation, (**d**) Elevated precipitation, and (**e**) N addition. Values (estimated from *lnRR_++_* in Equation (4)) represent mean effect sizes. Sample size numbers are shown in parentheses on the left. The error bars represent 95% confidence intervals (CI) and indicate a significant (*p* < 0.05) effect when not overlapping with 0. Note that the class “Total” includes all data from wetland, desert, forest, cropland, and grassland.

Considering the data of all land-use types, an elevated CO_2_ concentration generally increased the soil nutrient concentrations (Figure 2a). Across all the studies, the elevated CO_2_ concentration significantly increased the SOC by 4.1% (*p* < 0.05), which was consistent with previous meta-analyses [25,41]. The long-term differences between carbon inputs and the decomposition process determine the amount of SOC concentration in soil. In this case, an increase in SOC occurred because increasing atmospheric CO_2_ concentrations can increase the photosynthetic rate of plants and improve water use efficiency and nutrient use efficiency, which can reduce soil water loss through transpiration. These lead to increased plant productivity, plant litter, and roots, resulting in higher biomass C inputs into the soil and increased SOC content [42]. No significant change was observed in other soil nutrients. In the croplands, AK and TN were increased by elevated CO_2_ concentrations (2.2% and 3.8%), while the AN and AP were slightly decreased (−2.6% and −2.7%). In previous meta-analyses, Yue et al. [34] and Yue et al. [43] also found decreases in the AN and AP with elevated CO_2_ concentrations, and they thought the result might be because elevated CO_2_ concentration promotes plant growth and triggers a relatively larger uptake of N and P [44]. Comparing across the land-use types, the response of the SOC to elevated CO_2_ concentrations was not significant in wetlands (−0.5%) but was significant in cropland(+11.6%) (Figure 2a). The wetlands is a huge reservoir of organic carbon, and it is also the ecosystem with the fastest carbon accumulation rate. The persistence of this important ecosystem and its capacity as a carbon sink depends directly on the balance between organic matter production and decomposition in wetland soils. Enhanced decomposition will offset enhanced productivity and soil carbon accumulation in wetlands responding to elevated CO_2_ [45], which results in an insignificant decrease in soil SOC in wetlands.

#### 3.1.2. Effects of Warming

Except for TC and AN, warming had no significant effect on other soil nutrients. Overall, warming significantly decreased the TC by an average of 3.4% (Figure 2b) (*p <* 0.05), which is consistent with the findings of Yue et al. [25] and Zhou et al. [41], who also found that the soil TC and warming had the same pattern in a previous meta-analysis. Warming increased soil respiration rate and litter decomposition, resulting in C outflow. In addition, it inhibited the increase in plant root biomass and plant-derived C input, resulting in a decrease in TC content in soil [46]. In addition, warming also has a slight negative effect on soil SOC. An increase in air temperature may cause an increase in soil respiration and enhanced SOC decomposition because of increased microbial activity [42,47]. The AN (+15.2%) was significantly increased by warming, which was the most sensitive indicator to warming (Figure 2b) (*p <* 0.05). Yue et al. [43] also found warming had a significantly positive effect on the AN. Yue et al. [34] found that the TP significantly decreased with warming. However, in our meta-analysis, the TP was not significantly affected by warming. For SOC and TN, the impact of warming on forest and grasslands was smaller than that of croplands (Figure 2b). This may be because these experimental periods are relatively short, and the background of these indicators in forest and grassland soils is huge, which may not cause large changes in SOC and TN pools [48]. In both croplands and grasslands, warming had a great influence on AP. Short-term warming can regulate soil organic phosphate mineralization by directly changing the activity of soil phosphatase and indirectly changing the phosphorus demand of plants and microorganisms, thus regulating the phosphatase secretion, thus affecting the content of soil organic phosphorus [49]. Moreover, warming generated significant impacts on soil TK in the croplands (*p* < 0.05), leading to the remarkable increase by 5.1% (Figure 2b). Warming can promote the growth of plants and nutrient uptake, resulting in the transfer of nutrients to plants [50,51]; warming also tends to improve the beta diversity of soil microbial community and promote microbial growth [6,52], and their joint effects result in changes in the soil nutrient concentration.

#### 3.1.3. Effects of Precipitation

The effects of reduced precipitation and elevated precipitation on soil nutrients are shown in Figure 2c and Figure 2d, respectively. In the grasslands, precipitation significantly affect the soil AK, AN, and AP. The AK, AN, and AP were significantly increased by an average of 2.9%, 6.6%, and 10.6%, respectively, by reduced precipitation (*p* < 0.05). In contrast, elevated precipitation significantly decreased the AK, AN, and AP by 11.3%, 12.0%, and 3.9%, respectively (*p* < 0.05). This is because elevated precipitation increases the leaching of soil available nutrients and accelerates the loss of soil nutrients. Both reduced and elevated precipitation significantly decreased the TP by 3.4% and 8.5%, respectively (*p* < 0.05). He and Dijkstra [53] reported that drought significantly decreased P uptake to plants and thus resulted in the accumulation of TP in the soil. In addition to these significant effects, elevated precipitation increases the SOC by 5.2% (*p* > 0.05). Previous meta-analyses reported increased precipitation enhanced microbial activity, in the aspects of microbial biomass carbon [32], soil respiration [33,41,54], and enzymes activity [55]. The promoted microbial activity was considered as a possible cause of higher SOC contents in elevated precipitation cases.

#### 3.1.4. Effects of N Addition

Significant positive N addition effects (*p* < 0.05) on the TC (+4.6%), TN (+6.1%), SOC (+5.0%), and AN (+74.6%) were observed considering all the land-use types (Figure 2e). The positive effects of N addition on the TC also agreed with the findings from previous studies [25,56]. N addition also showed significantly positive effects on soil N pools, which is consistent with previous studies [43,57,58]. AN was the most strongly increased, especially in deserts (+158.9%) (Figure 2e). The reason for the result is apparently that N-containing compounds are directly added into the soil, mainly in the form of urea and NH_4_NO_3_ [24], and that a smaller soil AN pool than the TN caused more sensitive responses to environmental change drivers than the TN. TK was also increased by N addition (+12.4%), but the AK was decreased (−2.2%), and the effects were not statistically significant (*p* > 0.05). Considering the data of all land-use types, N addition had minor effect on TP and AP. Yue et al. [34] also found that N addition had no significant influence on the TP, but Deng et al. [59] reported a significant TP decrease with N addition. The reason for the different effects of N addition on the TP might be that P limitation and the underlying mechanisms varied among soil types, locations, or ecosystems. The TP was significantly decreased by N addition in deserts (−6.9%), significantly increased by N addition in croplands (+6.5%), and not significantly impacted in forests and grasslands. For desert and forest ecosystems, the N addition promotion on AP was positive, which is consistent with previous studies [30,34]. The reason might be that the soil AP was closely related to the mineralization of the organically bound P, which could be encouraged by N addition. TK (+14.45% and +8.55%, respectively) and AK (+5.46% and +1.50%, respectively) were increased in forests and grasslands, but in croplands, AK was significantly decreased (−14.73%). This may be a result of farmland K limitation following N fertilization to promote crop growth. Comparing across the land-use types, N addition has the greatest impact on wetlands. The previous literature reported that N fertilization has significant effect on soil organic matter, nutrient storage, and alkalinity [60]. It was possible that the addition of N had an effect on the salinity of wetlands, resulting in a greater effect of nitrogen addition on wetlands. Soil salinity was a key determinant for soil microbial communities in desert and coastal ecosystems, and soil organisms, available nutrients, microbial diversity, biomass, and metabolic activities decreased along the increase in salinity gradient. The reason lies in the fact that the decline of soil salinity inhibited the disintegration of soil aggregates, ammonia volatilization, nitrogen leaching loss, and SOC mineralization, whereas it increased the abundance, activity, and carbon and nitrogen assimilation potential of soil microorganisms [61].

### 3.2. Combinations and Interactions of Global-Change Factors on Soil Nutrients

**Figure 3:**
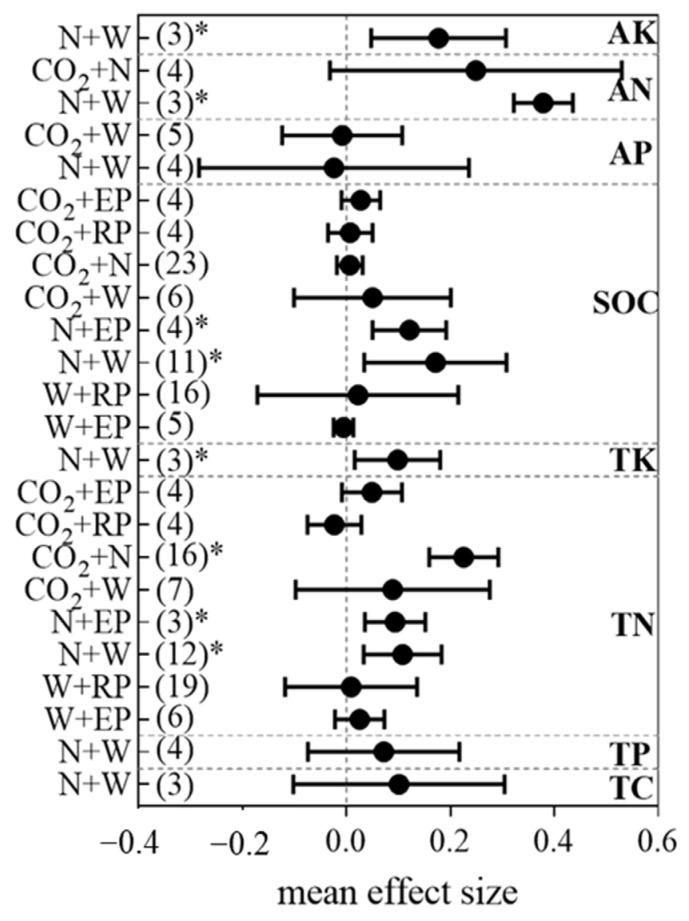
Combinations of climate change drivers on soil nutrients. Values (estimated from *lnRR_++_* in Equation (4)) represent mean effect sizes. The sample size numbers are shown in parentheses on the left. Level of significance (*p* < 0.05: “*”; 0.05 < *p* < 0.1: “ ”) is shown on the right. The error bars represent 95% confidence intervals (CI) and indicate a significant (*p* < 0.05) effect when not overlapping with 0. Results are not presented when sample size is lower than three.

**Figure 4:**
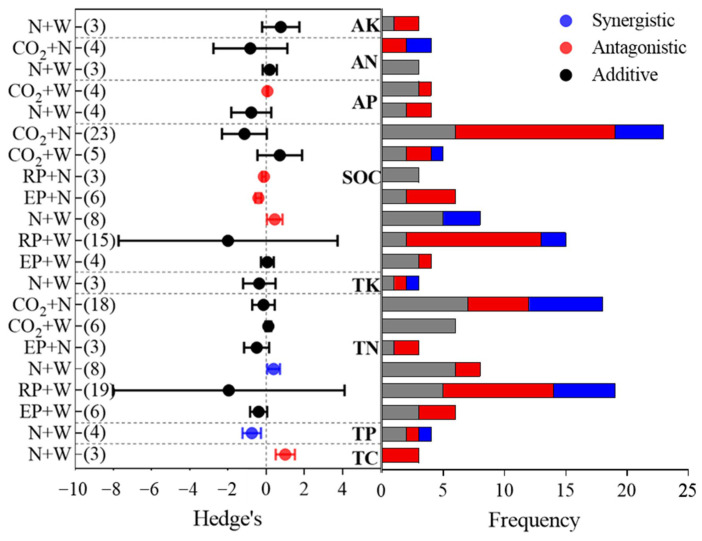
Interactions effects of multiple global change factors on soil nutrients and the frequency distribution of interaction types among individual observations of two-driver pairs. Values represent means with 95% confident intervals (CIs) and the numbers of sample size are shown in parentheses on the left.

Understanding the combinations and interactions of multiple global change factors on soil nutrients is vital for the prediction of soil nutrients, the correction of earth system models, and the development of management policies to future global changes. The combinations and interactions of global change factor pairs on soil nutrients are shown in Figure 3 and Figure 4, respectively.

Among all the driver pair interactions on the SOC, the combined effects of warming and N addition (N+W), N addition and reduced precipitation (N+RP), and N addition and elevated precipitation (N+EP) were antagonistic; the combination of warming and elevated precipitation (W+EP), warming and reduced precipitation (W+RP), elevated CO_2_ concentration and warming (C+W), and elevated CO_2_ concentration and N addition (C+N) were additive; the interaction of N+W on TC was also antagonistic (Figure 4). Here, we found that the SOC was significantly simulated by the combined effects of N+EP (+15.2%) and N+W (+17.7%) (Figure 3). Soil aggregates are the main sites of SOC fixation. It is estimated that about 90% of SOC in topsoils of terrestrial ecosystems is anchored in soil aggregates [62]. Added precipitation directly increases soil moisture, which may enhance biological processes that promote aggregate formation. However, nitrogen inputs negated the positive effects of increased precipitation on soil aggregate stability [63], and an antagonistic interaction was found for N+EP on the SOC. W+EP showed additive interaction effects on the SOC, which might be ascribed to positive microbe responses to warming and elevated precipitation [64], thus accelerating the decomposition of the SOC.

The combined N+W significantly increased the soil AN (+46.4%) and TN (+11.5%) (*p* < 0.05, Figure 3), which is consistent with the results from Yue et al. [43]; C+N and N+EP significantly stimulated a mean increase of 25.6% and 10.6% in the soil TN, respectively. N addition alone could significantly increase the soil TN, and further augmentation of the TN occurred when the N fertilization effect (i.e., N-induced increases in the plant N input to the soil) was enhanced by warming, elevated precipitation, or an elevated CO_2_ concentration [57]. The interaction of multiple drivers on the soil TN and AN are more likely to be additive, except that N+W showed synergistic interaction effects on the TN. Increased temperature generally stimulates biomass production and the uptake of limiting nutrients such as N. Moreover, warming is predicted to enhance soil microbial growth and their activities, stimulating N cycling [65]. Therefore, a synergistic interaction effect of N+W on the soil TN was observed.

Our results showed that the combination of N+W increased the TP (+3.6%) but decreased the AP (−8.9%), but the effects were not significant (Figure 3). The interactions of N+W on the TP were synergistic, which might be due to the same effect direction of the single driver of the pairs on vegetation, and mutual improvement occurred in the pairs. The C+W combination decreased the soil AP (−1.4%), and the interaction of C+W on the AP was antagonistic. The available driver pairs’ combined effects on the TP and AP were insignificant, and this finding might be due to the small sample sizes, which limit the statistical analyses [66].

The combination of N+W significantly increased the TK (+11.8%) and AK (+17.3%) (*p* < 0.05, Figure 3). The interactions of N+W on the TK and AK were both additive. K is reportedly controlled mainly by parent material weathering [9]. However, terrestrial ecosystems are biologically capable of retaining K through a variety of processes, such as the uptake of K by plants [67,68]. A review reported that N deposition increased K leaching in the soil, and the reduction in K concentrations and availability were also frequently observed [67]. K uptake by plants is closely related to soil moisture [69]. Warming affects the soil moisture, decreases K uptake, and affects soil microorganisms; overall significant positive effects of N+W on the TK and AK were observed in our study. The K concentrations in runoff and solutions are more sensitive than the N or P to environmental changes [70,71]. The leaching of K from litter is also quicker than that for N or P, and the residence time of K is shorter in soil organic matter [72].

### 3.3. The Role of Environmental Gradients

Moderator variables can significantly influence the individual and combined effects of global change factors on soil nutrients, and their effects were also reported by previous studies [24,25,33,34,43,59,73,74,75,76]. Geographical locations, climate factors, and experimental conditions are moderator variables impacting the soil nutrients’ responses to the effects of the investigated factors. In this study, the MAT, MAP, longitude, latitude, and experimental durations are important moderators that regulate soil nutrients under global changes. These factors can affect the response of soil nutrients to global change factors directly by regulating temperature and moisture conditions, or indirectly by intervening in microbial activity and vegetation (which is linked to soil nutrient cycling through plant uptake, leaching, mineralization, and decomposition rates) [77,78]. The experimental duration is usually vital for the assessment of ecosystems’ responses to global change drivers [79].

**Figure 5:**
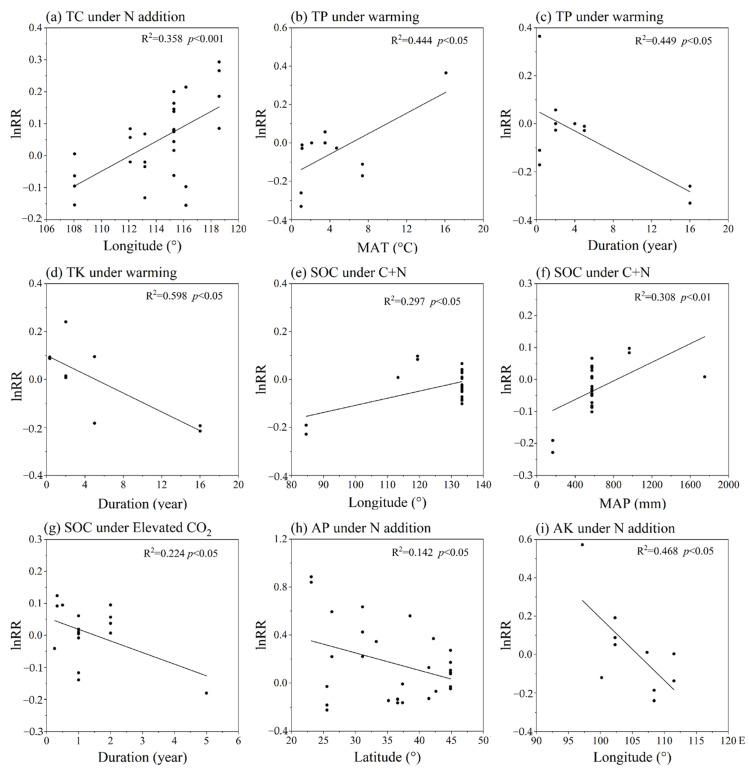
Correlations of the response ratios (*lnRR*) of soil nutrients with latitude, longitude, mean annual temperature (MAT), mean annual precipitation (MAP), and experimental duration to the individual and combined effects of multiple global change drivers. (**a**) correlations between lnRR of TC under N addition and longitude, (**b**,**c**) correlations between lnRR of TP under warming and MAT or experimental duration, (**d**) correlations between lnRR of TK under warming and experimental duration, (**e**,**f**) correlations between lnRR of SOC under C+N and longitude or MAP, (**g**) correlations between lnRR of SOC under elevated CO_2_ and experimental duration, (**h**) correlations between lnRR of AP under N addition and latitude, and (**i**) correlations between lnRR of AK under N addition and longitude. The condition for regression analysis was that the sample size was greater than five, thus the results were not available for some global change driver combinations. For simplification, only significant (*p* < 0.05) relationships are shown here.

The impacts of the moderator variables under different treatments are shown in Figure 5. The *lnRR* of TC induced by N addition displayed significant positive correlation with longitude (*p* < 0.05) (Figure 5a), indicating that TC of eastern areas (near the ocean) had more tendency to increase due to N addition than that of western areas (inlands). The warming induced *lnRR*(TP) was significantly positively correlated to MAT (Figure 5b), while warming induced *lnRR*(TP) and *lnRR*(TK) significantly negatively correlated to experimental duration (*p* < 0.05) (Figure 5c,d, respectively). The long-term warming is likely to cause the loss of soil TP and TK. The experimental duration also had a significant negative influence on the response ratios in SOC with elevated CO_2_ treatment (*p* < 0.05) (Figure 5g). Leuzinger et al. [80] considered that in the climate manipulation experiments of biosphere responses to global changes, a general trend for the magnitude of the responses to decline with higher-order interactions, longer time periods, and larger spatial scales occurred, and also might be due to plant and microbial acclimation under experimental treatments, and negative feedback mitigated the impacts [81,82,83]. The *lnRR* of available nutrients (AP and AK) induced by N addition treatment significantly decreased with increasing latitude and longitude (*p* < 0.05) (Figure 5h,i, respectively), that is, available nutrient concentration tended to increase with N addition in cold and dry regions (northeast China) but tended to decrease with N addition in hot and humid regions (southwest China). The C+N induced *lnRR*(SOC) significantly increased with increasing longitude and MAP (*p* < 0.05) (Figure 5e,f, respectively). MAP is generally higher in areas close to the ocean, and the longitude degrees of these areas are higher than that of inland areas in China. Therefore, Figure 5e,f reported the same result that SOC was more sensitive to C+N treatment in humid regions than in dry regions, implying that desertification due to increased drought may weaken the soil C sink in the future CO_2_-enriched and N deposition-increased world. SOC is one of the hot spots in soil nutrient research. Previous research has found that at higher elevation, the top SOC was high, which may be due to high clay content and low temperature. Increased use of chemical fertilizers leads to increases in top SOC, resulting in increased crop yields. Elevation, bulk density, and N fertilizers were the main factors controlling the top SOC [84].

### 3.4. Uncertainty Analysis, Limitations and Suggestions

We have synthesized the knowledge on how soil nutrients respond to the individual, combined, and interactive effects of multiple global change factors, and the results will help to predict future ecosystem changes and provide evidence and suggestions for perfecting earth system models. Significant uncertainty still remains due to the restrictions in the statistical methodologies and deficient experimental designs.

First, the observation-weighted approach applied may potentially overestimate the additive interactions associated with large variances in some observations. Second, our results showed that additive interactions are more frequent across all observations. This may be because the sample size of the two-driver pairs available is very small and therefore may hide potential synergistic or antagonistic effects. Third, the lack of three or more driver groups limited the breadth of the analysis. Additionally, vegetation and microbes play critical roles in soil nutrient cycling, but in the present meta-analysis, we focused on changes in soil nutrient concentrations but did not discuss how these elements changed in plants or microorganisms.

From the findings and limitations, we suggest the following: (1) There are few manipulative experiments that have three or more global change drivers, making it difficult to accurately analyze the interactions of more factors, so well-designed experiments that incorporate multiple factors of global change are in demand to generate multidimensional response surfaces to accurately quantify soil variable responses to future global change scenarios; (2) Most of the included studies investigated changes in the soil nutrients over less than five years; however, a longer duration for the environmental changes might alleviate the changes due to the compensation effects. Long-term studies should be undertaken to better understand the influences of global changes on soil nutrient cycling. (3) Future research could focus on potential biogeochemical mechanisms to enhance ecosystem stability to global changes. (4) The combined effects and their interactions with multiple drivers should be added to earth system models for accurate prediction of ecosystem changes.

## 4. Conclusions

Considering the vital roles of soil nutrients in element cycling and in terrestrial ecosystems, their responses to global changes would help to predict future ecosystem changes and to develop management policies. This synthesis showed that (1) among all the individual global change factors (N addition, warming, elevated CO_2_ concentration, and altered precipitation) which showed different individual effects on the soil nutrients, the soil nutrients were most sensitive to N addition; (2) the combined effects of driver pairs also varied among driver pairs and the soil nutrients, and the interactions of two drivers are most likely to be additive followed by antagonistic and synergistic; and (3) the experimental settings and environmental conditions (such as the location and the ecosystem type of the sites, experimental duration) also affected the responses of the soil nutrients to climate-change drivers. Multiple global change factors always occur simultaneously; therefore, future studies of soil nutrients should focus on long-term experiments and manipulative experiments with more global change drivers. When using earth system models to predict soil nutrients under global changes, taking the drivers’ combined and interactive effects and moderator variables into consideration would be recommendations for future research.

## Data Availability

Not applicable.

## Supplementary Materials

The following supporting information can be downloaded at: https://www.mdpi.com/article/10.3390/ijerph192215230/s1. Supplementary Materials: Dataset.
